# The Homeostatic Chemokine CCL21 Predicts Mortality and May Play a Pathogenic Role in Heart Failure

**DOI:** 10.1371/journal.pone.0033038

**Published:** 2012-03-12

**Authors:** Arne Yndestad, Alexandra Vanessa Finsen, Thor Ueland, Cathrine Husberg, Christen P. Dahl, Erik Øie, Leif Erik Vinge, Ivar Sjaastad, Øystein Sandanger, Trine Ranheim, Kenneth Dickstein, John Kjekshus, Jan Kristian Damås, Arnt E. Fiane, Denise Hilfiker-Kleiner, Martin Lipp, Lars Gullestad, Geir Christensen, Pål Aukrust

**Affiliations:** 1 Research Institute for Internal Medicine, Oslo University Hospital Rikshospitalet, Oslo, Norway; 2 Department of Cardiology, Oslo University Hospital Rikshospitalet, Oslo, Norway; 3 Section of Endocrinology, Oslo University Hospital Rikshospitalet, Oslo, Norway; 4 Department of Thoracic and Cardiovascular Surgery, Oslo University Hospital Rikshospitalet, Oslo, Norway; 5 Section of Clinical Immunology and Infectious Diseases, Oslo University Hospital Rikshospitalet, Oslo, Norway; 6 Institute for Experimental Medical Research, Oslo University Hospital Ullevål, Oslo, Norway; 7 Center for Heart Failure Research, University of Oslo, Oslo, Norway; 8 Faculty of Medicine, University of Oslo, Oslo, Norway; 9 Division of Cardiology, Stavanger University Hospital, Stavanger, Norway; 10 Faculty of Medicine, University of Bergen, Bergen, Norway; 11 Department of Cardiology and Angiology, Hannover Medical School, Hannover, Germany; 12 Department of Molecular Tumor Genetics and Immunogenetics, Max-Delbrück Center for Molecular Medicine, Berlin, Germany; I2MC INSERM UMR U1048, France

## Abstract

**Background:**

CCL19 and CCL21, acting through CCR7, are termed homeostatic chemokines. Based on their role in concerting immunological responses and their proposed involvement in tissue remodeling, we hypothesized that these chemokines could play a pathogenic role in heart failure (HF).

**Methodology/Principal Findings:**

Our main findings were: (i) Serum levels of CCL19 and particularly CCL21 were markedly raised in patients with chronic HF (n = 150) as compared with healthy controls (n = 20). A CCL21 level above median was independently associated with all-cause mortality. (ii) In patients with HF following acute myocardial infarction (MI; n = 232), high versus low CCL21 levels 1 month post-MI were associated with cardiovascular mortality, even after adjustment for established risk factors. (iii). Explanted failing human LV tissue (n = 29) had markedly increased expression of CCL21 as compared with non-failing myocardium (n = 5). (iv) Our studies in CCR7^−/−^ mice showed improved survival and attenuated increase in markers of myocardial dysfunction and wall stress in post-MI HF after 1 week, accompanied by increased myocardial expression of markers of regulatory T cells. (v) Six weeks post-MI, there was an increase in markers of myocardial dysfunction and wall stress in CCR7 deficient mice.

**Conclusions/Significance:**

High serum levels of CCL21 are independently associated with mortality in chronic and acute post-MI HF. Our findings in CCR7 deficient mice may suggest that CCL21 is not only a marker, but also a mediator of myocardial failure. However, while short term inhibition of CCR7 may be beneficial following MI, a total lack of CCR7 during long-term follow-up could be harmful.

## Introduction

Chronic heart failure (HF) is a disorder characterized by low-grade immune activation and inflammation, as evident by elevated circulating and myocardial levels of inflammatory cytokines and chemokines, such as tumor necrosis factor (TNF)α, interleukin (IL)-1β, monocyte chemoattractant protein (MCP)-1, and IL-8 [1]. Levels of these mediators may provide important prognostic information, and several experimental studies have also suggested a pathogenic role for inflammatory cytokines in HF [1], [2]. However, the inflammatory response in HF remains to be completely understood. Identification of the most important mediators of the inflammatory pathways that could be involved in the pathogenesis of HF as well as their mechanism of action are issues that need further elucidation.

The chemokines CCL19 and CCL21, acting through their common receptor, CCR7, are termed homeostatic due to their role in immune surveillance and regulation of leukocyte movement during homeostasis [3], [4]. This chemokine system is primarily thought to be involved in homing of naïve T cells and antigen-presenting dendritic cells to lymph nodes. However, more recent studies have revealed roles for CCR7 and its ligands in inflammation and T cell homing into non-lymphoid tissue as well as in regulatory T cells (Tregs) trafficking, implying a more complex role for CCR7 in immune responses [5], [6]. Moreover, recent studies suggest that CCR7 and its ligands are expressed in non-lymphoid cells such as fibroblasts, vascular smooth muscle cells (SMC), and endothelial cells, potentially being involved in vascular inflammation, cell proliferation, and matrix remodeling [7], [8], [9].

Based on their essential role in concerting immunological and inflammatory responses as well as their newly discovered involvement in tissue remodeling, we hypothesized that CCL19 and CCL21 may play a pathogenic role in HF. Here, we elaborated this hypothesis by clinical studies in patients with chronic HF and in patients with acute HF following myocardial infarction (MI), as well as in studies of CCR7 deficient mice in an animal model of post-MI HF.

## Methods

### Ethics

The clinical parts of this study were approved by the local ethical committee (Regional ethics committee of Helse Sør-Øst; Permit number S-05172) and conducted according to the ethical guidelines outlined in the Declaration of Helsinki for use of human tissue and subjects. Informed written consent was obtained from all subjects. All animal experiments were carried out in accordance with institutional guidelines, and conform to the Guide for the Care and Use of Laboratory Animals published by the US National Institutes of Health (NIH Publication No. 85-23, revised 1996) and was approved by the Norwegian National Animal Research Committee (permit of approval number STFDU2796).

### Patients with chronic HF – cross-sectional analysis

Patients with stable HF (n = 150) for >4 months in New York Heart Association (NYHA) functional class II-IV, on optimal cardiovascular treatment regimens, attending the Department of Cardiology at Oslo University Hospital Rikshospitalet, were consecutively included in the study (*Table 1*). Most of the patients were evaluated by standard right- and left-sided cardiac catheterization. The underlying cause of HF was classified as ischemic heart disease (IHD; n = 66, none with acute coronary syndrome during the past 6 months) or dilated cardiomyopathy (DCM; n = 84) based on disease history and coronary angiography. Control subjects were 20 sex- and age-matched apparently healthy individuals based on disease history and clinical examination, but none of the controls underwent hemodynamic or echocardiographic examination (*Table 1*). None of the controls were taking any medication.

**Table 1:** Characteristics of the study groups.

	CTR	Chronic HF	Acute HF	
	(n = 20)	(n = 150)	Losartan (n = 119)	Captopril (n = 117)
Age [years]	56 [43–69]	56 [58–82]	69 [69–89]	66 [51–88]
Male [%]	85	79	69	72
Etiology [IHD/DCM]	0/0	44/56	119	117
NYHA class [I/II/III/IV]	0/0/0/0	0/25/45/31	31/49/20/0	41/50/9/0
LV-EF [%]	N/A	29 [9–81]	40 [20–60]	38 [15–60]
Nt-proBNP [pmol/L]	5 [1]–[13]	208 [1–4139]	1120 [152–2981]	1293 [176–3622]
Creatinine [µmol/L]	84 [61–94]	87 [44–459]	89 [55–209]	89 [61–190]
Medication [%]				
Medication [%]:				
ACE inhibitor	0	71	0	100
Angiotensin II receptor blocker	0	20	100	0
β-blocker	0	80	78	72
Diuretics	0	71	71	75
Warfarin	0	42	20	17
HMG-CoA reductase inhibitors	0	43	61	64

Data are median and range or number or percentage of subjects. Controls were healthy age- and sex-matched subjects. ACE, angiotensin converting enzyme; CAD, coronary artery disease; DCM, dilated cardiomyopathy; HMG-CoA, hydroxymethylglutaryl coenzyme A; IHD; ischemic heart disease; LV-EF, left ventricular ejection fraction; N/A, not available.

### Patients with acute HF following MI – longitudinal analyses

The design and main results of the OPtimal Trial In Myocardial infarction with Angiotensin II Antagonist Losartan (OPTIMAAL) have previously been reported in detail [10], Briefly, 5,477 patients with acute MI complicated with HF during the acute phase were randomly assigned and titrated to a target dose of losartan (50 mg daily) or captopril (50 mg three times daily) as tolerated. Median randomization time was 3 days after MI, and patients were followed for a median of 2.7 years for mortality and morbidity endpoints. The present study was a prospectively designed multi-center sub-study of the main OPTIMAAL trial comprising 236 patients from six centers that was designed to analyze plasma/serum levels of inflammatory mediators [11]. Except for the study drugs (losartan versus captopril), there were no differences in medications between the two treatment groups (*Table 1*). In both sub-studies (i.e., cross-sectional and OPTIMAAL), blood sampling was collected based on a strict protocols for blood sampling and storage. Peripheral venous blood was drawn into pyrogen-free blood collection tubes without any additives and allowed to clot before centrifugation (1500 *g* for 10 minutes). All serum samples were stored at −80°C and thawed <3 times. In both sub-studies, patients with significant concomitant disease such as infection, malignancy, or autoimmune disorder were not included and none were receiving immunosuppressive drugs.

### Tissue sampling from human myocardium

Tissue samples from human failing myocardium were removed from still-beating hearts immediately on explantation from 29 patients with end-stage HF (NYHA class III or IV; left ventricular (LV) ejection fraction [LV-EF] <35%; 21 DCM, 10 IHD; age 47±3 years) undergoing cardiac transplantation. Control human LV tissue was obtained from subjects whose hearts were rejected as cardiac donors for surgical reasons (n = 5). The cause of death of donors was cerebrovascular accident, and none had a history of heart disease. The hearts from these subjects had been kept on ice for 1 to 4 hours before tissue sampling. In nine patients with advanced HF (NYHA class IV; 8 male, 1 female; age 29±5 years), LV tissue was sampled at the time of implantation and at the time of removal (heart transplantation) of a continuous-flow LV assist device (LVAD; VentrAssist, Ventracor Ltd, Chatswood, Australia). Average time on LVAD was 8±1.7 months. In both failing and non-failing myocardium, LV tissue samples were snap-frozen in liquid nitrogen, and stored at −80°C until use. None of the patients (failing and control myocardium) had significant concomitant disease such as infection, malignancy, or autoimmune disorder.

### Miscellaneous

Serum levels of CCL19 and CCL21 were measured by enzyme immunoassays provided from R&D Systems (Minneapolis, MN). Serum levels of N-terminal pro-brain natriuretic peptide (Nt-proBNP) and C-reactive protein (CRP) were determined as previously reported [11].

### Mouse model of experimental post-MI HF

C57BL/6 mice were purchased from Møllergaard (Møllergaard, Denmark). CCR7^−/−^ mice were backcrossed for at least 8 generations onto the C57BL/6 background [12], and bred at the Institute for Experimental Medical Research, Oslo University Hospital Ullevål. CCR7^−/−^ and wild type (Wt) mice were characterized after ligation of the left coronary artery (MI) or sham operation. MI was induced in 8 week old mice as described [13]. Sham-operated animals underwent the same procedure except ligation of the artery. Seven days or six weeks after surgery, the animals were anesthetized and ventilated before echocardiography was carried out, as described elsewhere [13]. Echocardiography was carried out using a VEVO 2100 (Visualsonics, Toronto, Canada) before the animals were sacrificed. Echocardiographic examinations were performed under standardized conditions with the animals in the supine position, spontaneously breathing 1.5% isoflurane and 98.5% O_2_ on a mask. Echocardiographic data were analyzed off-line using VEVO 2100 1.1.0 software from Visualsonics. Three representative cycles were analyzed and averaged. Two-dimensional (2D) images of the LV were obtained both in long and short axes. Short axis recordings were obtained at the level of the papillary muscle. M-mode tracings were recorded in the long axis at the level of the papillary muscles and the aortic valves, with 2D guidance. LV wall thickness and cavity dimensions were measured through the largest diameter of the ventricle both in systole and diastole. LV fractional shortening (LVFS) in percent, was calculated using the following formula: LVFS = (LVDd−LVDs)/LVDd×100 where LVDd is LV diameter in diastole, and LVDs is LV diameter in systole. Doppler recordings were obtained in the left parasternal long axis position. Pulsed wave Doppler was used for measuring flow velocities in the left ventricular outflow tract (LVOT) and in the mitral annulus. Cardiac output (CO) was calculated in LVOT using the following equation: CO = LVOT VTI×π×(diameter/2)^2^×heart rate where VTI is the velocity time integral and diameter is measured in LVOT. The animals were subsequently euthanized and their hearts were removed and blotted dry. The non-infarcted LV, infarcted area, right ventricular free wall, and lungs were weighed and normalized to tibia length. Based on previous findings [13], only mice with an infarction circumference >40% of total LV circumference, lung weights >133% of the average of the sham group, and a left atrium diameter >20 mm measured by 2D echocardiography were considered to have HF and were included in the post-MI HF group.

### Quantitative Real-Time RT-PCR

Total RNA from mouse and human myocardium was extracted using TRIzol (Invitrogen, San Diego, CA), DNase treated, cleaned up using RNeasy Mini Columns (Qiagen, Hilden, Germany), and stored at −80°C. cDNA was synthesized from 1 µg RNA using High Capacity cDNA Archive Kit (Applied Biosystems, Foster City, CA). Quantification of gene expression was performed using the ABI Prism 7500 (Applied Biosystems), 5 ng cDNA, Power SYBR Green Master Mix (Applied Biosystems), and sequence-specific PCR primers were designed using the Primer Express software, version 3.0 (Applied Biosystems). Primer sequences can be provided on request. Gene expression of the housekeeping gene GAPDH was used for normalization.

### Immunohistochemistry

Formalin fixed and paraffin-embedded mouse LV was immunostained with anti-CCL21 (diluted 1∶40 in PBS; R&D Systems), and anti-CD45 (diluted 1∶100 in PBS; Millipore, Bedford, MA). For forkhead box P3 (foxP3) analysis, acetone-fixed cryosections were stained with anti-foxP3 antibody (diluted 1∶100 in 1× PBS; eBiosciences, Cambridge, UK). Omission of the primary antibody served as negative control. The immunoreactivities were amplified by the avidin-biotin-peroxidase system (Vectastain Elite kit, Vector Laboratories) according to the manufacturers' instructions. Diaminobenzidine was used as the chromogen. The sections were counterstained with hematoxylin or eosin (foxP3). For the quantification of foxP3-positive cells, we randomly counted and averaged five fields (40× magnification) in the area bordering the myocardial infarction.

Masson trichrome staining of formalin-fixed and paraffin-embedded mouse hearts were performed using the Trichrome Stain (Masson) Kit (HT15; Sigma-Aldrich) according to the manufacturer's suggestions.

### Statistical analysis

If more than three groups were compared, the Kruskal-Wallis test was used *a priori*. For investigating treatment effects, repeated-measures ANOVA was performed *a priori* on log transformed values if necessary as evaluated by the Kolmogorov-Smirnov test, with time and treatment as fixed factors and subject as random. For comparisons of 2 groups of individuals, the Mann-Whitney U test was used. Coefficients of correlation were calculated by the Spearman rank test. Receiver-operating characteristics (ROC) curve were generated to evaluate the accuracy of each marker for prediction of all-cause and cardiovascular (CV) mortality. Kaplan-Meier analysis with log-rank test was performed to compare the number of events in different groups (comparisons pooled over strata). Cox proportional hazard analysis was performed to estimate hazard ratios using a forced method adjusting for age, hypertension, diabetes type 2, etiology, creatinine, CRP and Nt-proBNP. CCL21 was dichotomized (cross sectional study) or divided into tertiles with the two lower tertiles used as reference (OPTIMAAL). Follow-up time for all-cause mortality was calculated from time of inclusion to death from any cause. P values are two-sided and considered significant when <0.05. All analyses were performed with SPSS for Windows version 15.0 (SPSS, Chicago, IL).

## Results

### Serum levels of CCL19 and CCL21 are elevated and CCL21 levels predict mortality in patients with chronic HF

**Figure 1:**
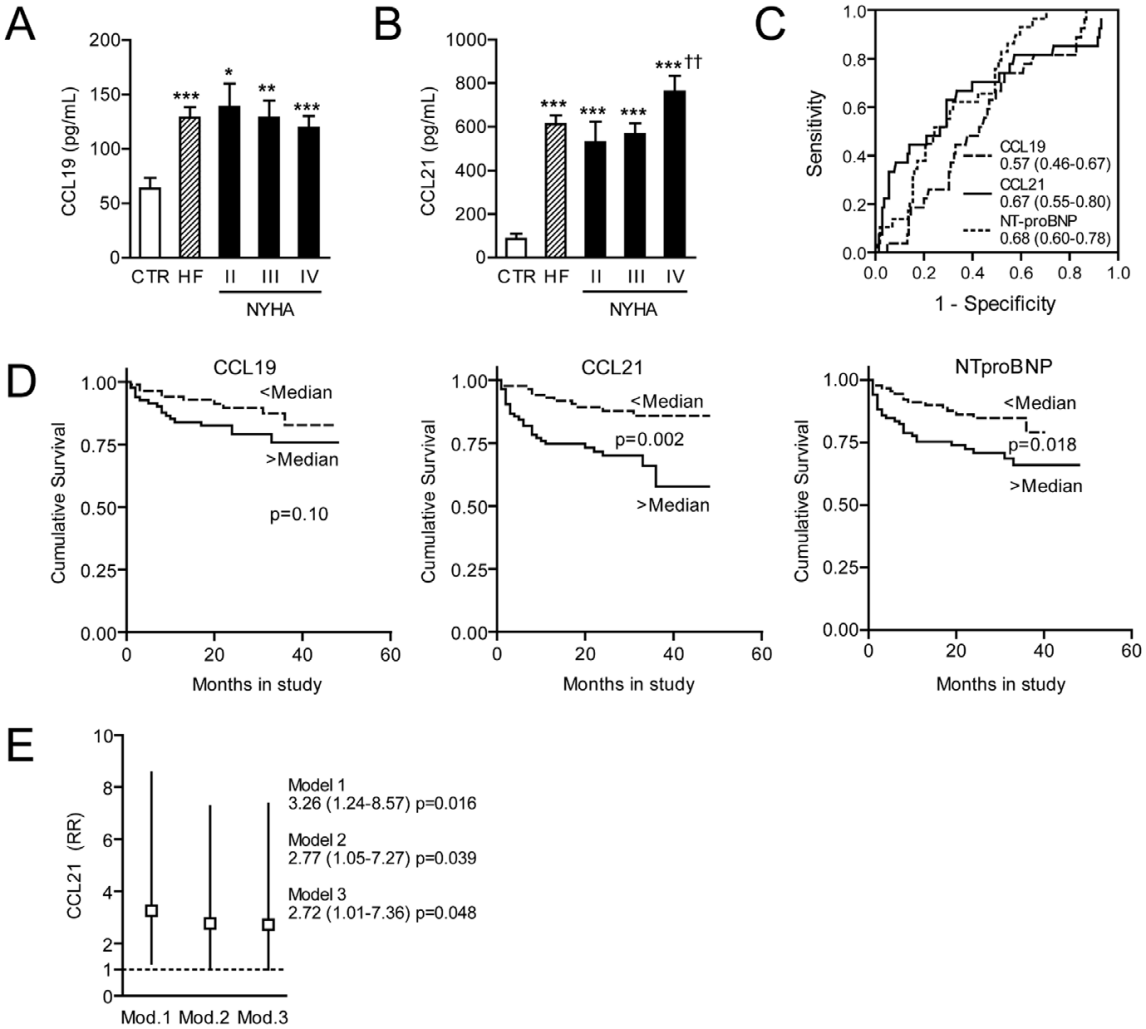
Serum levels of CCL19 and CCL21 are elevated and CCL21 levels predict mortality in patients with chronic heart failure (HF). The top panels show serum levels of CCL19 (**A**) and CCL21 (**B**) in patients with chronic HF (n = 150; NYHA class II/III/IV, n = 40, 71, and 39, respectively) and in 20 sex- and age-matched healthy controls, and (**C**) shows ROC curve analysis for the predictive value of CCL19, CCL21, and NT-proBNP for all-cause mortality during 24 months follow-up. AUC and 95% CI are given. The bottom panels show **(D**) Kaplan–Meier curves demonstrating the association between dichotomized serum levels of CCL19, CCL21, and NT-proBNP and incidence of all-cause mortality during 24 months follow-up, and (**E**) Cox-regression models adjusting for age and creatinine levels (Model 1), additional adjustment for LDL cholesterol, hypertension, diabetes type 2, and etiology (Model 2), and additional adjustment for CRP and Nt-proBNP (Model 3). Data are mean±SEM. *p<0.05, **p<0.01, and ***p<0.001 versus controls; ††p<0.01 versus NYHA class II and III.

As shown in *Figure 1A–B*, patients with chronic HF (n = 150) had significantly raised serum levels of CCL19 and CCL21 compared to healthy controls (n = 20). While CCL19 was not significantly different according to NYHA class, particularly high CCL21 concentrations were found in those with the most severe HF (*Figure 1A–B*). Moreover, CCL21, but not CCL19 was correlated to poor cardiac function as estimated by cardiac index (r = −0.35, p<0.001) and neurohormonal activation by means of Nt-proBNP (r = 0.39, p<0.001). Furthermore, patients with IHD (n = 66) had markedly elevated CCL21 compared to patients with DCM (n = 84) (747±66 pg/mL versus 494±46 pg/mL, p = 0.001), and notably, an opposite pattern was seen for CCL19 with the highest levels in DCM (129±10 pg/mL versus 154±97 pg/mL, p = 0.05). Importantly, however, both etiological sub-groups had significantly raised serum levels of CCL19 and CCL21 compared with healthy controls (data not shown). Levels of CCL21, but not CCL19, were significantly correlated with age in the HF patients (r = 0.37; p<0.001). Importantly, however, the difference between HF patients and controls was statistically significant also after adjusting for age (p<0.001 for both CCL19 and CCL21). In contrast to the interaction with age (CCL21), we found no interaction between gender and serum levels of CCL19 and CCL21 (data not shown).

During a mean follow-up of 24 (±12 [SD]) months, 32 patients died. ROC curves showed comparable area under curve (AUC) for CCL21 and NT-proBNP, with lower levels for CCL19 (*Figure 1C*). A combined ROC curve for CCL19 and CCL21 showed no discriminatory properties (AUC [0.56, 95% confidential interval [CI] [0.46–0.66]) as compared with ROC curves for CCL21 alone (*Figure 1C*).

As depicted in *Figure 1D*, CCL21 levels above median levels, but not CCL19 levels, were closely associated with all-cause mortality in this population. Similar to CCL21, high levels of NT-proBNP (>median levels) were associated with significant increased all-cause mortality during follow-up (Figure 1D). When comparing high versus low CCL21 concentrations in the total patient population, correcting for age and creatinine levels, the hazard ratio (HR) was 2.84 (1.39–5.82), p = 0.004 (*Figure 1E*, model 1). After additional adjustment for LDL cholesterol, hypertension, diabetes type 2, etiology (*Figure 1E*, model 2), and CRP and Nt-proBNP (*Figure 1E*, model 3), this relationship remained significant. Also, the cox-regression indicated that CCL21 alone was the only predictor when analyzed together with CCL19 alone and with the interaction term (data not shown).

### Serum levels of CCL21 predict mortality in patients with acute post-MI HF –results from the OPTIMAAL trial

**Figure 2:**
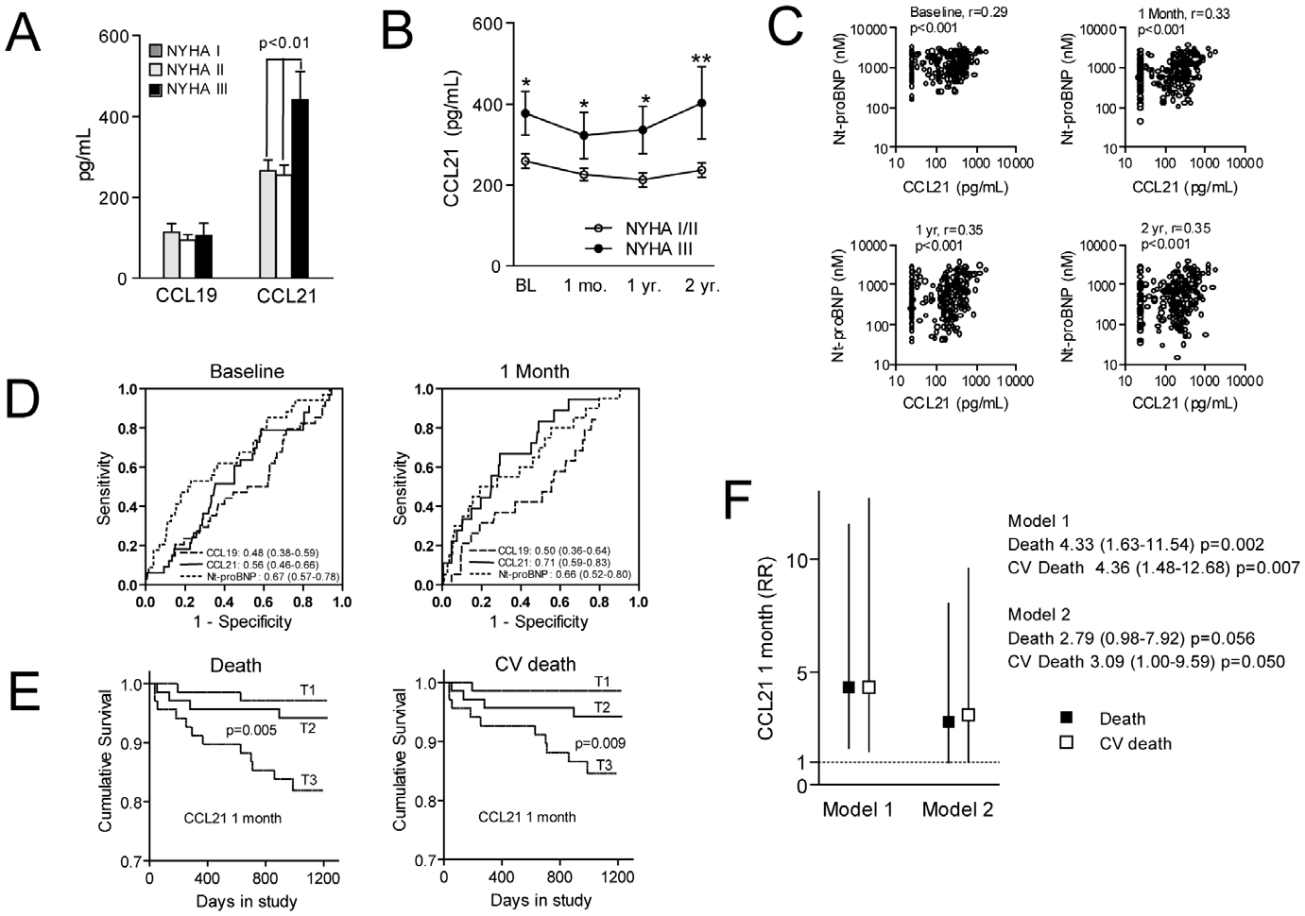
Serum levels of CCL21 predict mortality in patients with acute post-myocardial infarction (MI) heart failure (HF). Panels **A** and **B** show serum levels of CCL19 and CCL21 at baseline (BL; randomization time) (**A**) and in relation to NYHA functional class during follow-up (**B**) in a total of 236 HF patients. Data are mean±SEM. *p<0.05 and **p<0.01 versus NYHA class I/II. Panel **C** shows the correlation between serum levels of CCL21 and Nt-proBNP at baseline and at different time points during follow-up. Yr, year. Panel **D** shows ROC curve analysis for the predictive value of CCL19, CCL21, and NT-proBNP for all-cause mortality (n = 32) during 2.7 years follow-up at baseline and 1 months. AUC and 95% CI are given. Panel **E** shows Kaplan–Meier curves demonstrating the association between tertiles of serum CCL21 levels at 1 month and incidence of all-cause and CV mortality during 24 months follow-up. Panel **F** shows Cox-regression models for the tertile 3 of CCL21 versus tertile 1 and 2 combined either unadjusted (Model 1) or adjusted for age, creatinine levels, LDL cholesterol, hypertension, diabetes type 2, CRP and Nt-proBNP (Model 2).

At baseline, i.e., randomization time, significantly elevated levels of CCL21, but not of CCL19, were found in patients with NYHA class III as compared with those in NYHA class I/II (*Figure 2A*), and this difference persisted throughout the study period (*Figure 2B*). Moreover, at all time points, CCL21, but not CCL19, were significantly correlated with plasma levels of Nt-proBNP (*Figure 2C*). During an average follow-up of 27 months, there was no difference between the captopril and the losartan group in relation to total (n = 32) and CV (n = 26) mortality or in relation to serum levels of CCL19 and CCL21 (data not shown). ROC analysis showed that CCL21 levels, but not CCL19 levels, after 1 month, but not at baseline, were associated with total mortality and in particular CV death, with an AUC exceeding that of Nt-proBNP (*Figure 2D*). Kaplan-Meier plot showed a higher total mortality and CV death in patients in the highest CCL21 tertile as compared with the other patients (*Figure 2E*). When comparing high versus low CCL21 concentrations in the total patient population (highest versus two lower tertiles), the unadjusted HR was 4.33 (1.63–11.54); p = 0.002) and 4.36 (1.48–12.68); p = 0.007), total and CV death, respectively (*Figure 2F*, model 1). This relationship remained significant for CV death, but not for total mortality also after adjustment for age, creatinine levels, LDL cholesterol, hypertension, diabetes type 2, CRP and Nt-proBNP (*Figure 2F*, model 2).

### Myocardial CCL21 expression is increased in clinical and experimental HF

**Figure 3:**
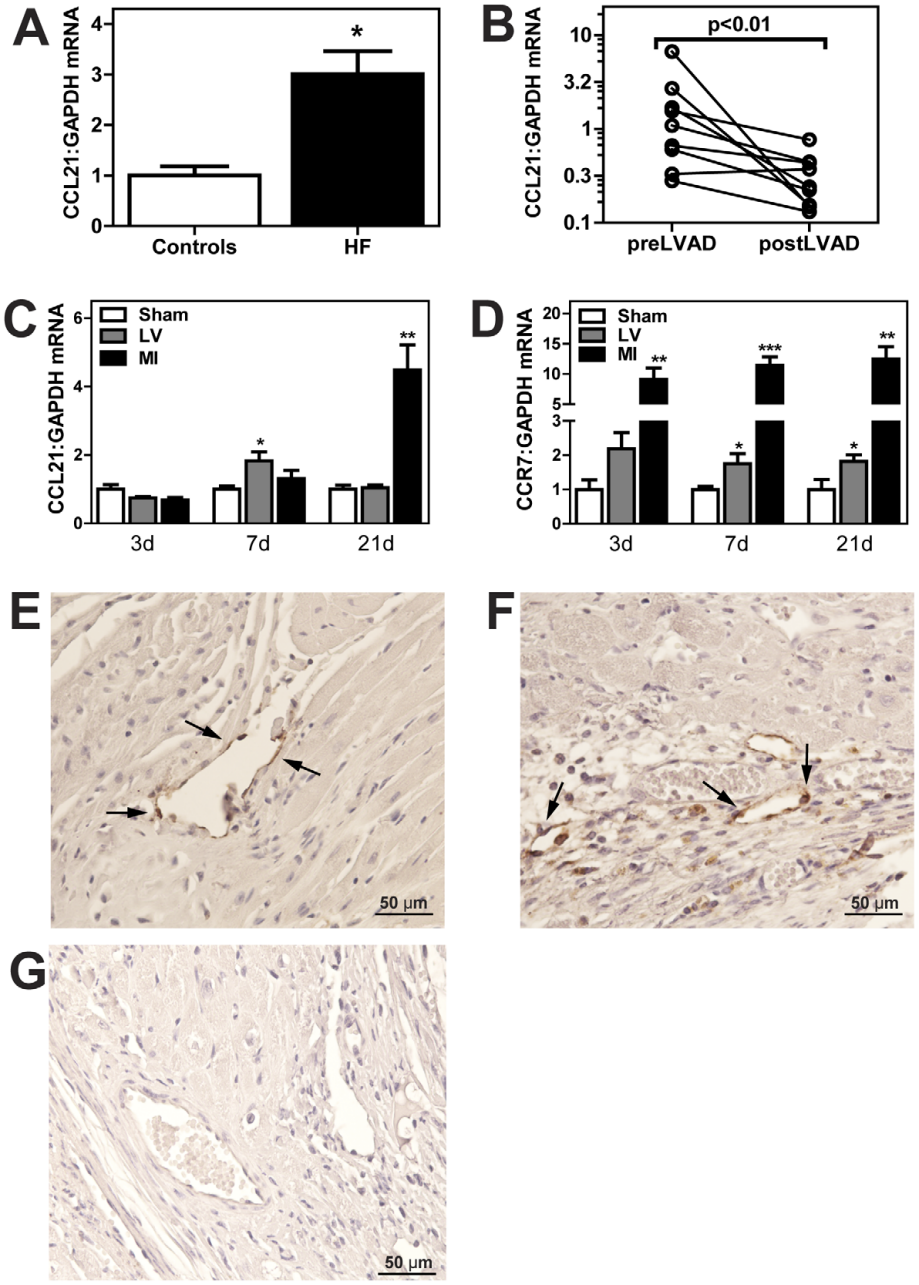
Myocardial CCL21 expression is increased in clinical and experimental heart failure. Gene expression of CCL21 in left ventricular tissue from (**A**) controls (donor hearts rejected for transplantation; n = 5) and explanted failing hearts (n = 29) and (**B**) heart failure patients (n = 9) during the implantation (preLVAD) and removal (postLVAD) of a continuous-flow LV assist device. The middle panels show gene expression of CCL21 (**C**) and CCR7 (**D**) in non-ischemic left ventricular tissue (LV) and infarcted area (MI) in mice 3, 7 or 21 days (d) after MI (n = 6–8) or sham operation (n = 6). mRNA levels were quantified by real-time RT-PCR and are presented relative to the gene expression of GAPDH. Data are mean±SEM. *p<0.05, **p<0.01 and ***p<0.001 vs. controls/sham. The bottom panels show immunohistochemical staining (magnification ×100) of CCL21 in LV from sham operated mouse (**E**) and mouse 1 week after MI (**F**) and a negative control image (**G**; omission of primary antibody). Arrows indicate CCL21-positive cells.

As shown in *Figure 3A*, the failing LV in 29 patients with advanced HF (NYHA class IV) had markedly increased expression of CCL21, but not of CCR7 and CCL19 (data not shown), as compared with the non-failing LV (n = 5) as assessed by real-time RT-PCR. Average C_t_ values for CCL21, CCR7, CCL19 and GAPDH were 27.3, 30.2, 30.7 and 18.8, respectively. In 9 of the patients, the improvement in hemodynamic and neurohormonal parameters that was seen during continuous-flow LV assist device (median follow-up time 8 months, range 1–18 months), was accompanied by a marked decrease in CCL21 mRNA levels, and this decrease was seen in 8 of the 9 patients (*Figure 3B*). Finally, we examined the myocardial expression of CCL21 and CCR7 in post-MI HF mice. In this experimental model of HF we found a significant myocardial up-regulation of CCL21 and CCR7 following MI. While persistently raised CCR7 levels were seen throughout the observation period (i.e., 21 days), CCL21 showed a more gradual rise with particular enhanced expression after 21 days (*Figure 3C–D*). Immunohistochemistry showed specific myocardial immunostaining of CCL21 in sham-operated and in particular in post-MI HF mice, with particularly strong immunostaining in endothelial cells (*Figure 3E–G*). Unfortunately, we were not able to obtain reliable immunostainings for CCR7.

### Improved survival in post-MI HF in CCR7^−/−^ mice

**Figure 4:**
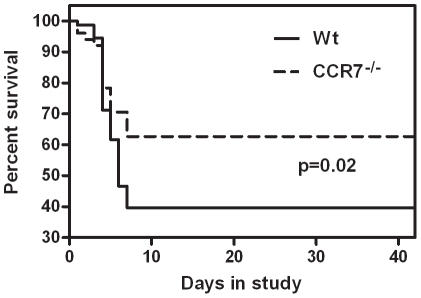
Improved survival in CCR7 deficient mice after myocardial infarction. Kaplan-Meier curve demonstrating increased survival in CCR7^−/−^ mice as compared to wild type mice after myocardial infarction. Differences in survival were tested with the log-rank test.

Our data so far may suggest activation of CCR7 in experimental and clinical HF. To elucidate potential functional consequences of these findings, we studied the effect of targeted disruption of CCR7 in a model of post-MI HF. As depicted in Kaplan-Meier survival curves, CCR7^−/−^ mice exhibited significantly higher survival rates than Wt mice during an 6 weeks follow-up after induction of post-MI HF (*Figure 4*). A total of 73 Wt and 51 CCR7^−/−^ mice were included. During follow-up 44 (60.3%) Wt and 19 (37.3%) CCR7^−/−^ mice died, reflecting improved survival in CCR7 deficient mice during the first week of follow-up (*Figure 4*).

### Expression of markers of myocardial function and fibrosis in post-MI HF in CCR7^−/−^ mice

**Figure 5:**
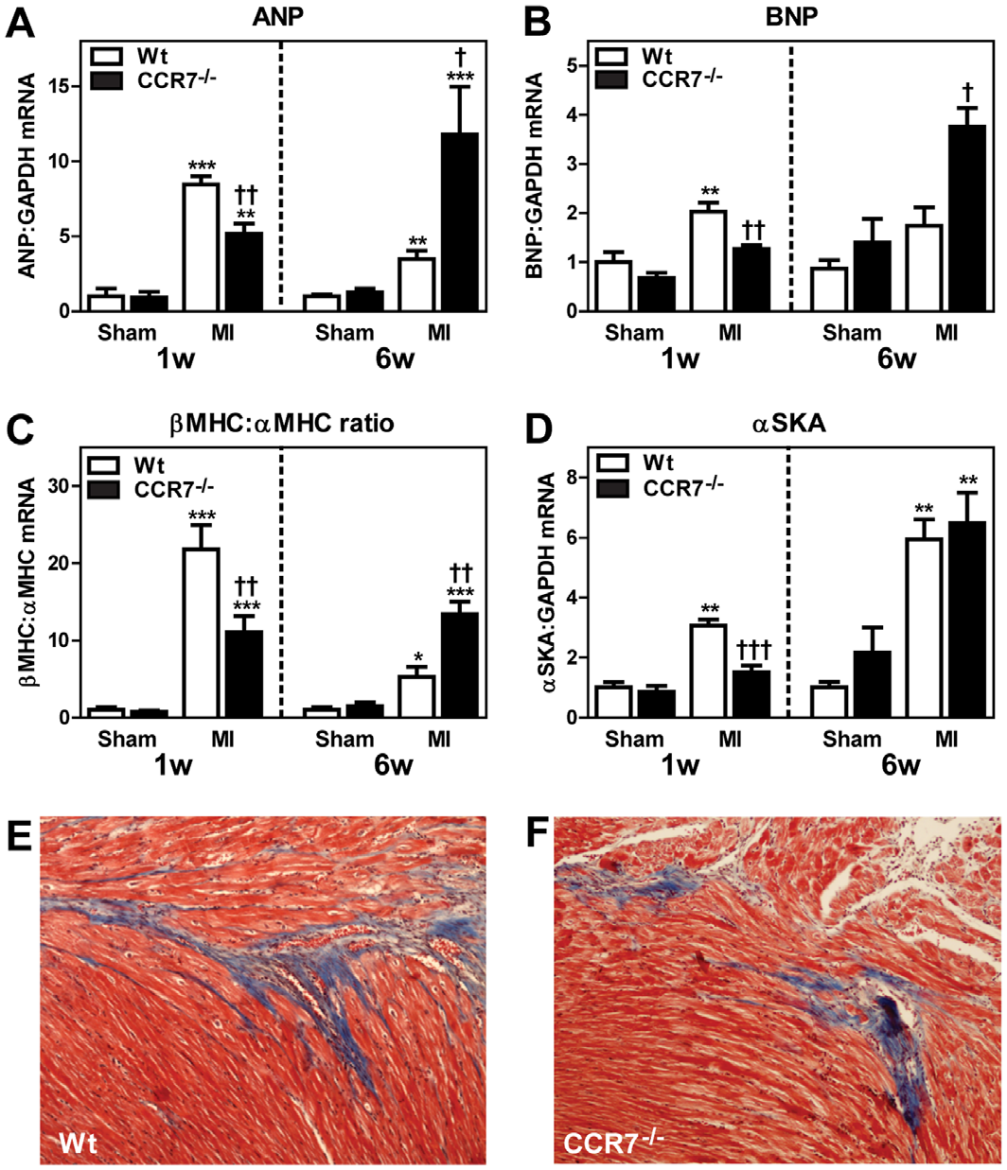
The myocardial expression of markers of myocardial hypertrophy and wall stress in CCR7^−/−^ and Wt mice one and six weeks post-myocardial infarction (MI). The panels show the gene expression of ANP (**A**), BNP (**B**), β-myosin heavy chain (MHC) to αMHC ratio (**C**), and α-skeletal actin (αSKA; **D**) in the left ventricle one and six weeks following sham operation or myocardial infarction (MI) in Wt (1w: n = 7+8; 6w: n = 5+7) and CCR7 deficient (1w: n = 6+9; 6w: n = 8+9) mice (numbers denote sham operation and post-MI HF, respectively). mRNA levels were quantified by real-time RT-PCR and are presented relative to the gene expression of GAPDH. Data are mean±SEM. *p<0.05, **p<0.01 and ***p<0.001 versus sham operation in the same genotype. †p<0.05, ††p<0.01 and †††p<0.001 versus Wt post-MI HF. Lower panels are representative images of Masson trichrome stained non-ischemic LV (magnification 10×) from Wt mouse (**E**) and CCR7^−/−^ mouse (**F**) six weeks after MI.

As shown in *Figure 5A–D*, the improved survival rate during the first week in CCR7^−/−^ mice was accompanied by an attenuated increase in the expression of ANP, BNP, α-SKA, and β-MHC/α-MHC ratio CCR7^−/−^ as compared with Wt mice following MI, potentially suggesting attenuated wall stress and improved myocardial function in CCR7 deficient mice. Somewhat surprisingly, an opposite pattern was seen 6 weeks following MI with increased ANP, BNP and β-MHC/α-MHC ratio expression in CCR7^−/−^ mice as compared with Wt mice (*Figure 5A–D*). As assessed by echocardiography, the differences between CCR7^−/−^ and Wt mice were modest and non-significant after 1 week (Table 2). However, the increase in myocardial expression of ANP, BNP and β-MHC/α-MHC ratio in CCR7^−/−^ mice 6 weeks after MI, was accompanied by an increase in LV diameter and a decrease in posterior LV wall thickness in diastole in these mice as compared with Wt mice, indicating increased LV dilation in CCR7 deficient mice at this time-point (*Table 3*). On the other hand, the degree of fibrosis as assessed by Masson trichrome staining in non-ischemic LV after 6 weeks showed no difference between sham-operated and post-MI HF mice, suggesting no or only minor differences in myocardial remodeling (*Figure 5E–F*).

**Table 2:** Animal characteristics and echocardiographic measurements in CCR7 deficient and wild-type mice 1 week after sham operation or myocardial infarction.

	Sham-Wt	Sham-CCR7^−/−^	MI-Wt	MI-CCR7^−/−^
	(n = 7–8)	(n = 7–8)	(n = 14)	(n = 14)
*Characteristics*				
BW, g	21.8±1.1	23.2±1.1	20.6±1.1	20.8±1.0
TL, mm	16.1±0.2	16.1±0.1	16.1±0.1	15.9±0.1
Heart rate, bpm	333±16	406±35	400±26	390±15
LVW, mg	76±2	74±1	93±1*	110±1*
INFW, mg	-	-	3.1±0.5	3.2±0.3
RVW, mg	19±2	20±3	19±0.3	27±0.2
LW, mg	145±2	137±3	214±19*	274±16*
*Echo-MM*				
Inf. circ., mm	-	-	10.8±0.7	11.5±0.7
LVDd, mm	3.7±0.2	3.3±0.1†	4.8±0.1*	5.1±0.1*
PWd, mm	0.9±0.04	0.9±0.03	0.8±0.04*	0.8±0.04
LVFS, %	24±2	22±2	12±1*	11±1*

Sham, 9–11 week old sham-operated group; MI, 9–11 week old myocardial infarction group; WT, wild-type mice; CCR7^−/−^, CCR7 knock-out mice; BW, body weight; TL, tibia- length; bpm, beats per minute; LVW, left ventricular weight; INFW, infarction weight; RVW, right ventricular weight; LW, lung weight; Echo-MM, M-mode echocardiography; Inf. circ, infarct circumference; LVDd, left ventricular diameter in diastole; PWd, posterior wall thickness in diastole; LVFS, left ventricular fractional shortening; ^*^p<0.05 vs sham in respective genotype group. Values are means±SEM.

**Table 3:** Animal characteristics and echocardiographic measurements in CCR7 deficient and wild-type mice 6 weeks after sham operation or myocardial infarction.

	Sham-Wt	Sham-CCR7^−/−^	MI-Wt	MI-CCR7^−/−^
	(n = 7–8)	(n = 7–8)	(n = 7–8)	(n = 10–11)
*Characteristics*				
BW, g	28.3±0.6	27.3±1.0	30.3±0.6	28.9±0.8
TL, mm	16.9±0.2	17.2±0.2	17.1±0.1	17.4±0.1
Heart rate, bpm	425±17	409±21	428±21	499±17
LVW, mg	90±2	98±4	124±6*	139±5*
INFW, mg	-	-	42±3	45±4
RVW, mg	24±1	25±1	32±2	35±2
LW, mg	148±31	191±18	197±12*	243±27*
*Echo-2D*				
LV circ/Inf. circ, %	-	-	51±2	57±3
*Echo-MM*				
LVDd, mm	4.1±0.2	4.5±0.09	6.4±0.2*	7.0±0.02*†
PWd, mm	0.81±0.06	0.80±0.03	0.86±0.05*	0.71±0.04†
LVFS, %	22±2	20±1	9±2*	8±1*
EF, %	43±4	41±2	20±3*	21±3*

MI, myocardial infarction; WT, wild-type mice; CCR7^−/−^, CCR7 knock-out mice; BW, body weight; TL, tibia- length; bpm, beats per minute; LVW, left ventricular weight; INFW, infarction weight; RVW, right ventricular weight; LW, lung weight; Echo-2D, two-dimensional echocardiography; LV circ/Inf. circ, left ventricular circumference/infarct circumference; Echo-MM, M-mode echocardiography; LVDd, left ventricular diameter in diastole; PWd, posterior wall thickness in diastole; LVFS, left ventricular fractional shortening; EF, ejection fraction; ^*^p<0.05 vs sham in respective genotype group and ^†^p<0.05 vs Wt in same group. Values are means±SEM.

### Increased myocardial expression of Foxp3, IL-10 and transforming growth factor (TGF)-β1 during post-MI HF in CCR7 deficient mice

**Figure 6:**
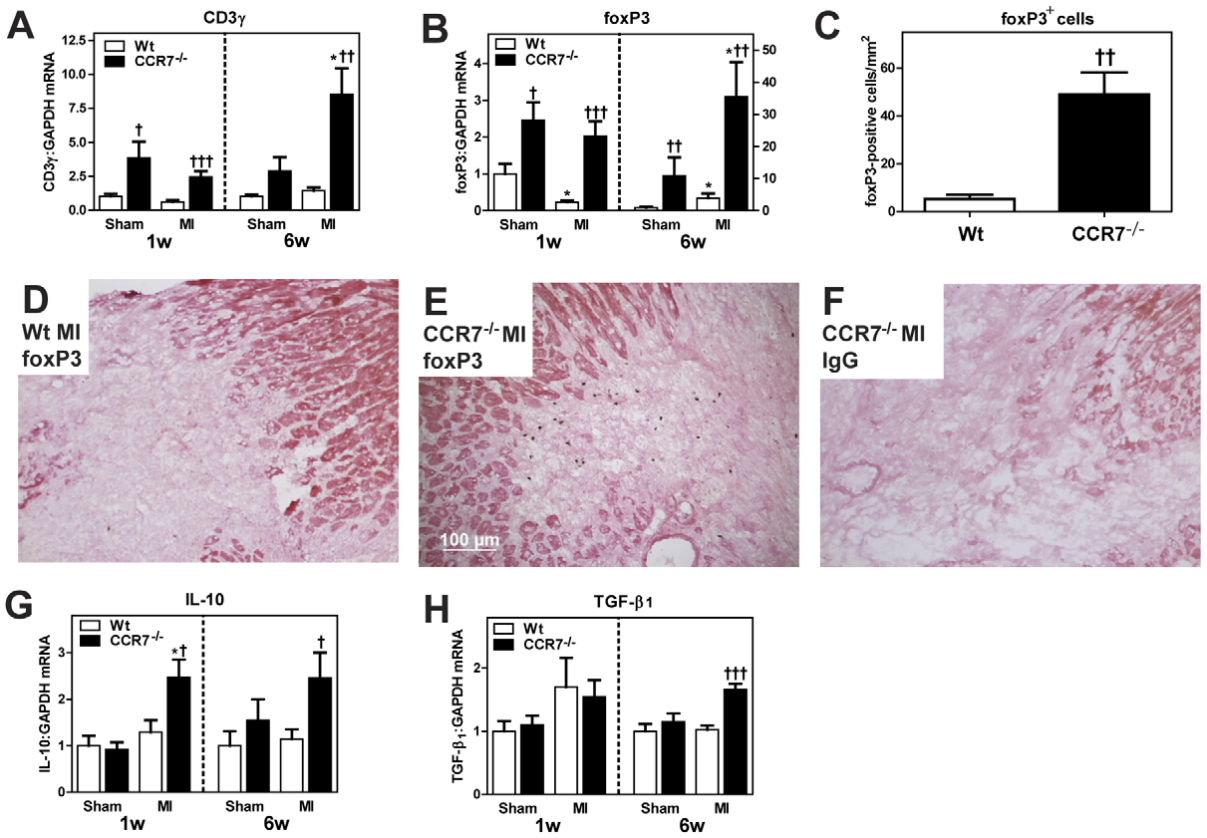
Increased myocardial expression of foxp3 and IL-10 in post-myocardial infarction (MI) heart failure (HF) in CCR7 deficient mice. Gene expression of CD3γ (**A**), foxP3 (**B**), IL-10 (**G**) and TGF-β_1_ (**H**) in LV one and six weeks following sham operation or MI in Wt (1w: n = 7+8; 6w: n = 5+7) and CCR7 deficient (1w: n = 6+9; 6w: n = 8+9) mice (numbers denote sham operation and post-MI HF, respectively). mRNA levels were quantified by real-time RT-PCR and are presented relative to the gene expression of GAPDH. Data are mean±SEM. Panel **C**. show average number of foxP3 positive cells in the area bordering the ischemic zone, and panels **D** and **E** show representative images of immunohistochemical staining of foxP3 in LV tissue from a Wt mouse (**D**) and a CCR7^−/−^ mouse (**E**) 1 week after MI. Panel **F** shows staining with the corresponding isotype control IgG antibody. *p<0.05 versus sham in same genotype; ^†^p<0.05, ^††^p<0.01 and ^†††^p<0.001 versus Wt post-MI HF.

**Figure 7:**
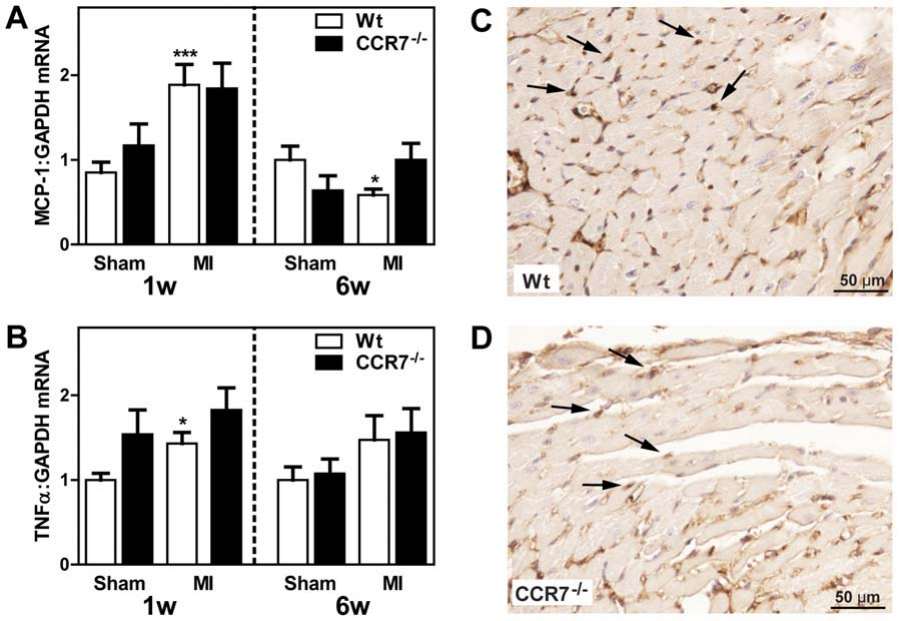
Myocardial gene expression of inflammatory markers in wild type and CCR7 deficient mice during post-MI HF. Left sided panels show gene expression of monocyte chemoattractant protein (MCP)-1 (**A**) and tumor necrosis factor (TNF)α (**B**) in the LV one and six weeks following sham operation or MI in Wt (1w: n = 7+8; 6w: n = 5+7) and CCR7 deficient (1w: n = 6+9; 6w: n = 8+9) mice (numbers denote sham operation and post-MI HF, respectively). mRNA levels were quantified by real-time RT-PCR and are presented relative to the gene expression of GAPDH. Data are mean±SEM. *p<0.05, and ***p<0.001 versus sham operation in the same genotype. Right sided panels show representative images of immunohistochemical staining of CD45 as a pan-leukocyte marker (magnification 40×) in LV tissue from a Wt mouse (**C**) and a CCR7^−/−^ mouse (**D**) one week post-MI. Arrows indicate CD45-positive cells.

As shown in *Figure 6A*, mRNA levels of CD3, a pan-T cell marker, were significantly increased in both sham-operated and post-MI HF CCR7^−/−^ mice after 1 week. CCR7 has been implicated in the regulation of Tregs trafficking [6], and mRNA levels of Foxp3, a sensitive marker for Tregs, exhibited markedly different pattern in the two types of mice. Whereas Foxp3 expression was significantly decreased in post-MI HF as compared with sham operated Wt mice after 1 week, the myocardial expression of Foxp3 was markedly enhanced in CCR7^−/−^ mice with no difference between sham operated and HF mice, potentially reflecting a phenotypic characteristic of these mice (*Figure 6B*). Also, immunohistochemistry showed strong Foxp3 immunostaining within the LV of post-MI CCR7^−/−^ mice as compared with LV from post-MI Wt mice (*Figure 6C–F*). IL-10 and TGF-β have been related to the function of Tregs [14], and the increased expression of Foxp3 in CCR7^−/−^ mice was accompanied by a marked increase in IL-10 in post-MI HF, but not of TGF-β_1_, as compared with Wt mice (*Figure 6G–H*). A similar pattern was seen after 6 weeks in the post-MI model with increased expression of CD3, Foxp3 and IL-10 in CCR7^−/−^ as compared with Wt mice (*Figure 6A, B and G*). However, while there was no increase in TGF-β_1_ in CCR7^−/−^ post-MI HF mice after 1 week, the myocardial expression of TGF-β_1_ in these mice after 6 weeks was markedly increased (*Figure 6H*). In contrast to the difference in Tregs related markers, mRNA levels of inflammatory cytokines (i.e., TNFα and MCP-1; *Figure 7A–B*) and CD45 immunostaining as a marker of leukocyte infiltration (*Figure 7C–D*), showed no differences between the two genotypes.

## Discussion

CCR7 and its ligands have previously been related to various inflammatory disorders such as rheumatoid arthritis, inflammatory bowel disease, and atherosclerosis [8], [15], [16]. Our data suggests that this chemokine system also could be involved in the development of HF. For the CCR7 ligand CCL21, markedly enhanced expression was found both systemically and within the failing myocardium in human HF. Moreover, high serum levels of CCL21 were independently associated with increased mortality in both chronic HF and acute HF following MI. Our studies in CCR7^−/−^ mice showed improved survival and attenuated increase in markers of myocardial dysfunction and wall stress in post-MI HF after 1 week. However, an opposite pattern was seen after 6 weeks with increased ANP, BNP and β-MHC/α-MHC expression in post-MI CCR7^−/−^ mice as compared with Wt mice. Although there was no increased mortality in CCR7^−/−^ mice after the first week, these latter findings may suggest impaired myocardial function during long-term follow-up in CCR7 deficient mice. Thus, while short-term inhibition of CCR7 signaling may be beneficial following MI, a total lack of CCR7 during long-term follow-up could be harmful, illustrating the fine balance between adaptive and maladaptive effects of inflammatory and anti-inflammatory mediators in post-MI remodeling and development of HF. Moreover, while enhanced expression of CCL21 as in clinical HF may be harmful, a total *and* persistent lack of its receptor, CCR7, may also have harmful effects on myocardial remodeling.

A wide range of inflammatory markers are elevated during HF such as TNFα, IL-6, IL-1, and various inflammatory chemokines [1]. The present study, however, is the first report of elevated serum levels of homeostatic chemokines in HF patients. We previously showed increased levels of CCL19 and CCL21 in coronary artery disease [8], and it may be argued that the raised levels of these mediators in HF merely reflect that several of these patients also have accompanying atherosclerosis. However, although patients with ischemic etiology had particularly elevated CCL21 levels, also patients with DCM had raised serum CCL21 levels compared with controls. Moreover, the ability of serum levels of CCL21 to predict survival in both chronic HF and in acute post-MI HF, even after adjustment for several confounders such as age, hypertension, GFR, CRP, and Nt-proBNP as well as the etiology of HF, may further suggest the involvement of CCR7 activation in the progression of HF. The significant decrease in the myocardial expression of CCL21 following hemodynamic and neurohormonal improvement during continuous-flow LV assist device treatment in advanced HF, gives additional support to a link between high CCL21 expression and impaired myocardial performance.

In contrast to CCL21, CCL19 showed no association with mortality in HF patients and no myocardial increase in advanced HF. The different association between CCL19 and CCL21 and parameters of HF may have several not-mutually exclusive explanations. First, while CCL19 is produced by several types of cells such as T cells, monocytes and macrophages, CCL21 seems primarily to be produced by stromal cells [3]. It is possible that the ability of CCL21 to predict mortality may reflect its regulation in stromal-related cells within the myocardium. Second, CCL21 has been found to induce more potent inflammatory effects in macrophages than CCL19 [8], potentially implicating a more important role in certain inflammatory disorders. Finally, although both chemokines are acting through CCR7, they could still mediate, at least in some degree, different effects. In fact, we have data showing that CCL19 and CCL21 differently affect both macrophages and vascular smooth muscle cells (Aukrust P, Yndestad A, unpublished data).

Decreased Tregs have been related to various autoimmune diseases, and appear to be involved in the pathogenesis of some CV disorders (e.g., atherosclerosis and heart allograft rejection) [14], [17], [18]. Recently, transfer of Tregs into angiotensin II-infused hypertensive mice was shown to improve cardiac hypertrophy despite sustained hypertension [19]. Based on these and the present data, it is tempting to hypothesize that Tregs also could be involved in the pathogenesis of HF. During post-MI HF there was a significant decrease in the myocardial Foxp3 expression in Wt mice, as a marker of Tregs. In contrast, the myocardial expression of Foxp3 was markedly enhanced in CCR7^−/−^ mice in both sham-operated and HF mice, potentially representing a phenotypic characteristic of these mice. It has previously been shown that CCR7 deficiency promotes the accumulation of Treg subsets in inflamed sites, accompanied by an enhanced suppression of the inflammatory reaction, suggesting a role for CCR7 in Tregs trafficking [5]. Following MI, CCR7^−/−^ but not Wt mice showed marked up-regulation of myocardial IL-10, a Treg-related cytokine that has been shown to inhibit post-MI LV remodeling [20]. However, a similar pattern was also seen after 6 weeks when the myocardium in CCR7^−/−^ mice showed increased levels of markers of myocardial dysfunction and wall stress. The reason for this pattern is not clear. However, it is possible that while a short-term increase in Tregs and IL-10 following MI, when Wt mice showed decreased Foxp3 expression, is adaptive, persistently increased levels may be maladaptive. In fact, the disappointing results of IL-10 therapy in inflammatory disorders may reflect that long-term administration may induce maladaptive responses, including enhanced B cell activation [21]. However, further studies are needed to elucidate the regulation and potential role of Tregs during myocardial remodeling.

The present study has some limitations such as the inclusion of relatively few patients and in particular, the number of controls was low. Also, the lack of IHC from human heart, the lack of IHC on CCR7, and the lack of data on serum levels of CCL19 and CCL21 in the mice model are other limitations. Moreover, the changes in echocardiographic variables were rather modest. However, mRNA markers of myocardial remodelling showed profound alterations, suggesting that these may be more robust markers of myocardial dysfunction in this model. The association between the attenuated up-regulation of these markers and improved survival further support such a notion. Finally, systemic deletion of CCR7 has some confounders, and future studies should also examine the effect of cardiac-restricted deficiency of CCR7. Nonetheless, our studies, combining experiments in clinical and experimental HF, suggest a role for CCL21/CCR7 interactions in the pathogenesis of HF.
